# Characterization of the Response of Magnetron Sputtered In_2_O_3−x_ Sensors to NO_2_

**DOI:** 10.3390/s23063265

**Published:** 2023-03-20

**Authors:** Enza Panzardi, Nicola Calisi, Nicoleta Enea, Ada Fort, Marco Mugnaini, Valerio Vignoli, Anna Vinattieri, Mara Bruzzi

**Affiliations:** 1 Department of Information Engineering and Mathematics, University of Siena, Via Roma 56, 53100 Siena, SI, Italy; 2 Dipartimento di Ingegneria Industriale, Università degli Studi di Firenze, Via S. Marta 3, 50139 Firenze, Italy; 3 INSTM, Consorzio Interuniversitario Nazionale per la Scienza e Tecnologia dei Materiali, 50019 Sesto Fiorentino, FI, Italy; 4 Dipartimento di Fisica e Astronomia, Università degli Studi di Firenze, Via G. Sansone 1, 50019 Sesto Fiorentino, FI, Italy; 5 Istituto Nazionale di Fisica Nucleare-INFN, Florence Section Via G. Sansone 1, 50019 Sesto Fiorentino, FI, Italy; 6 National Institute for Laser, Plasma and Radiation Physics, 077125 Magurele, Romania

**Keywords:** NO_2_ sensors, MOX gas sensor, In_2_O_3_ gas sensor, magnetron sputtering deposition

## Abstract

The response of resistive In_2_O_3−x_ sensing devices was investigated as a function of the NO_2_ concentration in different operative conditions. Sensing layers are 150 nm thick films manufactured by oxygen-free room temperature magnetron sputtering deposition. This technique allows for a facile and fast manufacturing process, at same time providing advantages in terms of gas sensing performances. The oxygen deficiency during growth provides high densities of oxygen vacancies, both on the surface, where they are favoring NO_2_ absorption reactions, and in the bulk, where they act as donors. This n-type doping allows for conveniently lowering the thin film resistivity, thus avoiding the sophisticated electronic readout required in the case of very high resistance sensing layers. The semiconductor layer was characterized in terms of morphology, composition and electronic properties. The sensor baseline resistance is in the order of kilohms and exhibits remarkable performances with respect to gas sensitivity. The sensor response to NO_2_ was studied experimentally both in oxygen-rich and oxygen-free atmospheres for different NO_2_ concentrations and working temperatures. Experimental tests revealed a response of 32%/ppm at 10 ppm NO_2_ and response times of approximately 2 min at an optimal working temperature of 200 °C. The obtained performance is in line with the requirements of a realistic application scenario, such as in plant condition monitoring.

## 1. Introduction

Due to the increase in anomalous events related to climate, with rising global temperatures and increasingly unpredictable weather events, monitoring air pollutants that can contribute to climate change has become of utmost importance. Nitrogen dioxide (NO_2_) is one of the most widespread and dangerous pollutants present in the atmosphere [1]. It is extremely harmful to humans and can be equally destructive to the environment since it contributes to the formation of ground-level ozone, a potent greenhouse gas that can harm plants and ecosystems. Moreover, NO_2_ is also a major component of acid rains.

NO_2_ originates from various sources, such as automobile exhaust and industrial emissions, and in general from all activities or plants involving combustion processes.

For these reasons, detecting harmful NO_2_ has emerged as a very important environmental monitoring strategy and the realization of responsive, cost effective and easily manufactured sensors is an urgent need [2].

Semiconducting metal oxides (MOXs) are among the most widely used materials for toxic gas sensing. Their peculiar chemical composition, morphology and nanosized structure allow for high surface area to volume ratios, making them particularly suitable for the development of resistive gas sensors, for which the surface chemical reactions are the base working principle [3,4,5,6]. MOX gas sensors are widely used in a wide range of fields, such as environmental control and monitoring, the automotive and food industry and clinical and biomedical applications [7,8,9,10].

The wide diffusion of MOX materials for the realization of chemoresistive gas sensors is due both to their chemical characteristics and to the technology involved in sensor manufacturing. Their benefits include low cost and easy manufacturing methods, simplicity of application by end users and an ability to detect a wide range of gas species. On the other hand, this sensor technology still faces limitations connected to gas cross-sensitivity and high-power consumption due to the high working temperature of the sensors [3,4,5,6].

Among the different methods employed for depositing gas sensing MOX thin films, magnetron sputtering [11] is a reliable and environmentally clean technique. It allows for manufacturing sensing layers with tailored morphologic characteristics, such as grain size, porosity and surface roughness, by opportunely tuning deposition parameters as oxygen (O_2_) flow ratio, power and working pressure, deposition time and substrate bias voltage [11,12,13].

Moreover, compared with other thick film traditional deposition processes, such as spin coating or drop casting and screen or ink jet printing [14], thin films obtained by magnetron sputtering methods are characterized by a much higher binding force and reproducibility [15,16,17], very good stability and they are suitable for the integration in miniaturized, low-power sensing devices based, for instance, on micro hot plates [18].

As far as NO_2_ detection is concerned, a wide range of metal oxides showed suitable sensing features, among which are SnO_2_, WO_3_, ZnO and In_2_O_3_ in their pure form, modified by doping or decoration and in composites. Some recent comprehensive reviews discuss this subject, comparing the performance reached by a plethora of MOX sensing layers [19,20,21]. Many different MOX sensors show excellent characteristics, especially in terms of sensitivity, reaching values higher than to 100%/1 ppm (defining the sensor response as the relative variation of the resistance in the presence and absence of NO_2_), and many of these sensors show good sensitivity also at room temperature, but usually resorting to light activation, which is extremely power-hungry, or to the addition of organic materials [19,22]. In general, the best performances are obtained exploiting nanostructured materials as nanorods, nanoparticles and nanoflowers that are often prepared in the form of powders and used as the basis of suspensions, inks or pastes, which are then deposited on sensor substrates by means of traditional thick film preparation techniques. These latter techniques compare unfavorably to thin film deposition processes, as discussed earlier [14]. Recently, it has been demonstrated that a novel method for enhancing the response toward NO_2_ and reducing the operating temperature relies on the introduction of defects, especially oxygen vacancies, into semiconductors [19,23]. This approach has the huge advantage of no need of any additional modification of the material, and further steps in the production process are not required [19]. It was shown that, for ZnO_1-x_-, SnO_2-y_-, WO_3-z_- and CuO_1-k_-based sensors, either response, recovery times or sensitivity improve with respect to the stoichiometric sensors [19,24,25].

Limiting the scope to NO_2_ sensors based on thin films, those prepared exploiting In_2_O_3_ show very good performances, as they exhibit a high sensitivity to oxidizing gases such as NO_x_, even at low working temperatures, and particularly to NO_2_ [12,26,27,28,29].

In this paper, we propose an In_2_O_3_-based sensor for the detection of low concentrations of NO_2_. The active layer is produced by a radiofrequency (RF) magnetron sputtering deposition process at room temperature and in an oxygen-free environment. The deposition technique allows for a facile and fast manufacturing of the film, ensuring high control of its morphological characteristics and excellent adhesion to the substrate [13,15,16]. Deposition in the absence of oxygen guarantees the possibility to reduce the complexity of the sputtering procedure and to induce oxygen vacancies, which allows for obtaining thin films with electrical resistance values in the kilohms range [17] (several available MOX gas sensors show baseline resistance in the order of megaohms). This represents an ideal condition for the development of the front-end electronics and of the measurement setup, ensuring better performance in terms of stability and resolution and the use of cost-effective systems. The lower values of the film resistance are basically due to the greater availability of oxygen bulk vacancies [23], and hence to the higher charge carrier concentrations (i.e., higher conductivity). In addition, oxygen starvation positively affects the sensitivity of the sensor to NO_2_, since it causes a higher availability of surface oxygen vacancies [20,23]. The surface absorption reaction of NO_2_ is then boosted, thus ensuring large responses even at low gas concentrations [23,29].

As far as the sensitivity of the film is concerned, another important aspect directly influencing the sensitivity of the film is its morphology. In this regard, Karthikeyan et al. in [30] and Li et al. in [12] extensively demonstrated how the oxygen content in the sputtering gas during the deposition process changes the grain size and surface roughness of the film. In particular, a decrease in the surface roughness and, conversely, an increase in the average particle size is observed with the decrease in the O_2_ flow.

In the case of the sensors realized in this work, the dependence of gas sensitivity on the surface roughness (high value of roughness provides large sensing surface area) is dominated by the roughness of the substrate, so the adverse effect of oxygen lack on the film roughness can be neglected.

The NO_2_ sensing performances of In_2_O_3−x_ sensors were characterized at different operating temperatures and in the presence of either rich or oxygen-free atmospheres.

### Sensing Mechanism

In_2_O_3_ is a transparent semiconducting material with a fundamental bandgap with E_g_ ~3.7 eV; it is typically cubic bixbyite with high crystalline quality [31,32]. Commonly, In_2_O_3_ shows an inherent n-type conductivity, and the high conductance values are attributed to the presence of oxygen vacancies and/or indium interstitials [32]. The sensor response of In_2_O_3_, as for all MOXs materials, is due to a change in its electrical conductance caused by target gas adsorption and desorption, accompanied by an exchange of electrons between the solid interface and the gas phase [33,34,35]. For an n-type semiconductor such as In_2_O_3_, a decrease in free carrier concentration is expected when exposed to oxidizing gases, as in the case of NO_2_. Indeed, the large changes in conductivity are explained by the existence of a depleted region of free carriers at the surface of the grains forming the film. The depleted region is caused by the trapping of free electrons by intrinsic defects (i.e., intrinsic electronic surface states) and by the molecules absorbed from the gas phase (extrinsic surface states). Therefore, its depth depends on the amount of adsorbed gas molecules. The depleted region is a charge space region, in which an electric field is established. Accordingly, a potential barrier exists at each grain boundary, opposing the electron conduction from one grain to the neighbor, which limits the inter-grain conduction. The variation of the potential barrier height due to the chemosorption of the target gas is usually the major phenomenon determining the conductivity of grain-sized sensing films. Conduction in such films results, therefore, in a thermally activated phenomenon described by an exponential law, where the activation energy is due to the potential barrier at the grain boundaries, variable with gas concentration. This explains the very large sensitivity and non-linear behavior of such sensing materials [33].

When the In_2_O_3_ sensing film is exposed to NO_2_ molecules, these are absorbed on the film and gain electrons from the surface according to the following reaction [12,26]:

(1) $$NO_{2\left(gas\right)}+S_{NO_{2}}+e^{-}\to \;NO_{2}^{-}{}_{\left(ads\right)}$$

where *e*^−^ indicates a free electron and $S_{NO_{2}}$ a site of absorption for NO_2_; in the considered case, most of the adsorption sites could be represented by In_2_O_3_ surface oxygen vacancies. This reaction describes the trapping of free electrons at the surface species, or in other words the creation of occupied extrinsic states, which gives rise to the increase in the depletion region depth at the surface of the indium oxide grains with a consequent increase in the resistance of the film [33,34,36].

If the sensing film is exposed to the target gas in the presence of air, the interaction of oxygen with the surface must also be taken into account.

In general, when the In_2_O_3_ surface is exposed to air, the oxygen molecules adsorb on its surface, creating extrinsic surface states, which can capture electrons from the conduction band of In_2_O_3_. The process involves different reactions, that can be described in the following general way [37,38,39,40]:

(2) $$\frac{\beta }{2}O_{2\left(gas\right)}+S_{O_{\beta }}+\alpha e^{-}\;\to O_{\beta }^{-\alpha }{}_{\left(ads\right)}$$

where *α* and *β* are natural numbers and can take the values one and two. In general, for many different metal oxides, among which is also In_2_O_3_, [12] for low temperatures (lower than a temperature which lies between 150 °C and 200 °C) the most probable reaction is the one with *β* = 2 and *α* = 1, corresponding to molecular chemisorption; at higher temperatures (in the range 150–400 °C), the oxygen molecule tends to dissociate and ionize (ionosorption). Therefore, *β* = 1 and *α* = 1; finally, at very high temperatures (>400 °C) the reaction with *β* = 1 and *α* = 2 is favored.

Due to the operating working temperature used in this study, both $O_{2}^{-}$ chemisorbed and $O^{-}$ ionosorbed species must be taken into account, related to the following adsorption reactions:

(3) $$O_{2\left(\mathrm{gas}\right)}+S_{O_{2}}+e^{-}\to O_{2}^{-}{}_{\left(\mathrm{ads}\right)}\;(\mathrm{dominant}\;\mathrm{for}\;\mathrm{temperatures}\;<150\;{}^{\circ}C/200\;{}^{\circ}C)$$

(4) $$O_{2\left(gas\right)}+S_{O_{2}}+e^{-}\to O_{2}^{-}{}_{\left(ads\right)}\;(\mathrm{dominant}\;\mathrm{for}\;\mathrm{temperatures}\;<150\;{}^{\circ}C/200\;{}^{\circ}C)$$

Thus, according to Equations (3) and (4), when the In_2_O_3−x_ film is exposed to a mixed atmosphere of NO_2_ and oxygen, the $O_{2}^{-}{}_{\left(ads\right)}$ or $O_{\left(ads\right)}^{-}$ species tend to take the place of NO_2_, probably in the same adsorption sites. This represents a competitive adsorption between oxygen and NO_2_ that may cause a reduction of the sensing response of the In_2_O_3_ sensor in the presence of air with respect to the one obtained with an inert carrier gas, e.g., pure nitrogen, N_2_ (i.e., in the absence of oxygen).

**Figure 1:**
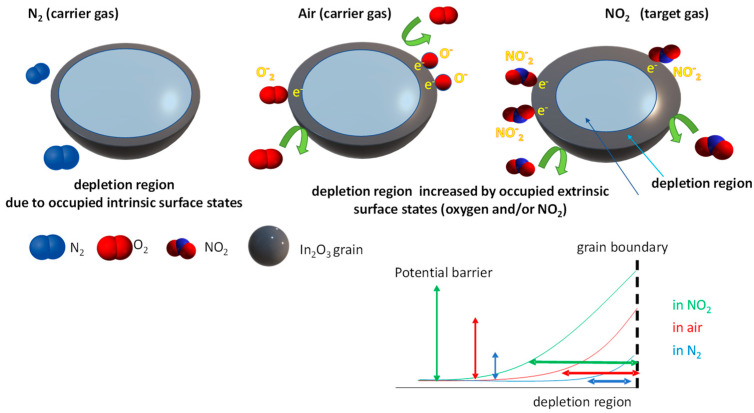
Gas absorption mechanism in In_2_O_3_ nano-grained material.

The discussed reaction routes and sensing mechanism are represented in Figure 1.

A further cause of a modification of the sensor response to NO_2_ in the presence of oxygen with respect to the one in its absence is related to the possible interaction of the two species. In particular, a reduction of the response can be due to the reaction of chemisorbed oxygen with chemisorbed NO_2_ [41]:

(5) $$2NO_{2\left(ads\right)}^{-}+O_{2\left(ads\right)}^{-}\;\to \;2NO_{3}^{-}{}_{\left(ads\right)}+e^{-}$$

On the other hand, the negative effect on the NO_2_ response due to the presence of oxygen could be partially compensated by another reaction reported in the literature [12], which involves preabsorbed oxygen in the NO_2_ adsorption as follows:

(6) $$NO_{2\left(gas\right)}+O_{2\left(ads\right)}^{-}+2e^{-}\;\to \;NO_{2}^{-}{}_{\left(ads\right)}+2O_{}^{-}{}_{\left(ads\right)}$$

In any case, a difference in the sensor sensitivity to NO_2_ measured in an inert environment or in air (i.e., in the presence of oxygen) is expected.

## 2. Sensing Film Deposition and Sensor Preparation

### 2.1. Sensor Preparation

The chemoresistive In_2_O_3_ sensors were deposited on alumina substrates 2 mm thick, 15 mm × 8 mm size and a surface roughness of approximately 800 nm [42]. The substrate was screen printed on both sides; one side held a heater, whereas the other one embedded the electrodes for the sensing film together with a screen-printed platinum resistance temperature detector (Pt-RTD) [42,43].

### 2.2. Film Deposition

The deposition of indium oxide (In_2_O_3_) thin films was performed by RF magnetron sputtering with pulse frequency 13.56 MHz starting from a 99.99% stoichiometric sintered In_2_O_3_ target. During a specific deposition, different substrates were coated to perform both gas sensing devices and specimens for material analyses. In particular, glass and quartz substrates were used for structural, optical and electrical characterization. The manufactured thin films, with a thickness in the range 150 nm to 500 nm, were grown at room temperature (RT), with a constant total pressure in the 10^−3^ mbar range, in Ar plasma atmosphere with no oxygen inlet and with a constant deposition rate of 5 Å/s.

## 3. Sensing Material Characterization

**Figure 2:**
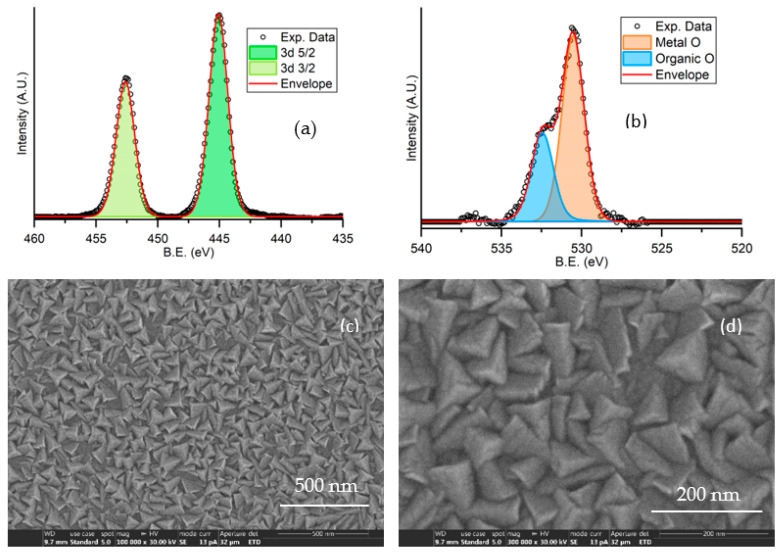
Characterization of an In_2_O_3−x_ 500 nm thick film deposited on a glass substrate: XPS spectra showing peaks for indium (**a**) and oxygen (**b**); SEM analysis at 10^5^ (**c**) and 3 × 10^5^ (**d**) magnification.

The as-obtained thin films were characterized by X-ray photoelectron spectroscopy (XPS), X-ray diffraction (XRD) and scanning electron microscopy (SEM). Moreover, optical transmission, four-point probe resistivity and Hall effect measurements were performed. The XPS analysis was performed with an X-ray source (VSW Scientific Instrument Limited model TA10, Al Kα radiation, 1486.6 eV). Results are shown in Figure 2a,b. The peak areas relative to indium and oxygen resulted in an almost 1:1 ratio, evidencing a stoichiometry in significant deficiency of oxygen. For this reason, the deposited film will be considered in the following as In_2_O_3−x_.

The film morphology was investigated by SEM analysis. Figure 2c,d show the SEM images with different magnification, evidencing the polycrystalline nature of the film, which consists of a compact and uniform distribution of grains with pyramidal shape and size in the range 50–100 nm.

**Figure 3:**
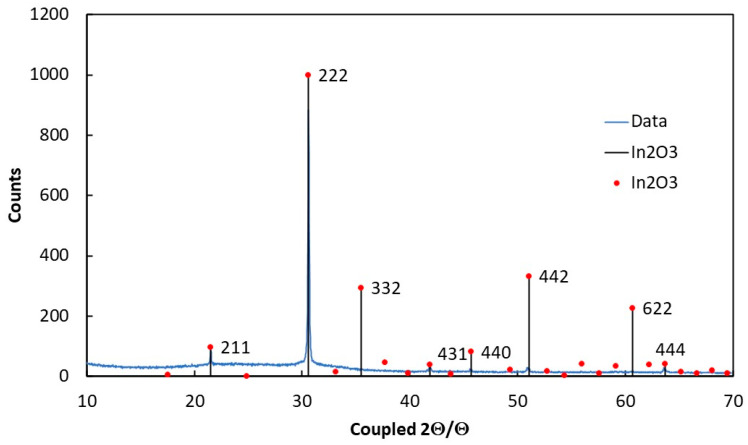
XRD spectrum of In_2_O_3−x_ on a glass substrate: comparison with peaks in cubic centered form BCC c-In_2_O_3_ (JCPDS card number 06-0416).

To perform a structural analysis of the deposited films, XRD measurements were carried out using a Bruker model D8 Advance diffractometer. The spectrum reported in Figure 3 shows the XRD analysis of the investigated sample and the literature of indium oxide (III) card, in body centered cubic BCC form crystalline c-In_2_O_3_ (JCPDS card number 06-0416). In agreement with previous studies on In_2_O_3−x_ samples, the highest peak intensity is measured for the (222) orientation [44].

From our XRD data, it is possible to calculate the lattice constant:

(7) $$\frac{1}{d^{2}}=\frac{h^{2}+k^{2}+l^{2}}{a_{0}^{2}}$$

where d is the distance between the adjacent planes in the set (hkl). In our case, the above equation was applied for the set of (222) planes, which have the highest intensity, with d = 0.91 nm. We obtained a value of the lattice constant, a_0_ = 1.008 nm, in fair agreement with the literature, *a* = 1.0118 nm [44,45].

**Figure 4:**
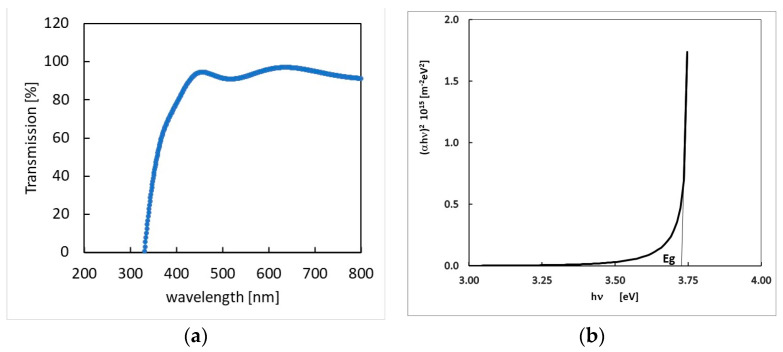
(**a**)—optical transmission spectrum of the In_2_O_3−x_ 500 nm thick sample; (**b**)—function ${\left(\alpha h\nu \right)}^{2}vs\;h\nu$ plot and linear fit used to determine the forbidden gap.

An evaluation of the grain dimension was carried out by fitting the highest peak corresponding to the 222 orientation. The grain size D is calculated by means of Scherrer’s formula:

(8) $$D=\frac{0.9\;\lambda }{B\cos\theta_{B}}$$

where *λ* = 0.154 nm and B is the measured broadening of the diffraction line peak at the angle 2θ at half its maximum intensity. The average crystal size in our films was determined to be in the range 70–80 nm by a Gaussian fit of the 222 peak and then using Equation (8). This value is almost equal to the grain size as from SEM images, evidencing the crystalline nature of the grains. The average crystal size found in our samples was more than double the size than that for InO_x_ prepared by DC magnetron sputtering and same rate deposition [44,46]. The optical transmission, T(ν), of a In_2_O_3−x_ film deposited on a quartz substrate as a function of the wavelength is shown in Figure 4a. From data, the absorption coefficient of the film as a function of the wave frequency, *ν*, can be obtained:

(9) $$\alpha \left(\nu \right)=\frac{1}{t}ln\left(\frac{1}{T\left(\nu \right)}\right)$$

where t = 500 nm is the thickness of the sample. For direct optically allowed inter-band transitions, the following relationship holds [46]:

(10) $$\propto \;\frac{1}{\nu }\sqrt{h\nu -E_{g}}$$

where *h* is the Planck constant. Therefore, the band-gap energy E_g_ can be estimated by linearly fitting the function ${\left(\alpha h\nu \right)}^{2}\;vs\;h\nu$ (see plot in Figure 4b).

The as obtained energy gap value, E_g_ = 3.73 eV, is in agreement with the literature [26,36].

Squared samples were deposited on glass substrates to determine the transport properties, resistivity, conductivity type and mobility of our films by four-point probe analysis of resistivity and Hall effect at room temperature. The measurement setup consisted of a Keithley 220 as current source and a Keithley 6517 as current readout, a Keithley 2182 nanovoltmeter for voltage readout and a Keithley 705 scanner for switching among different electrode configurations. The resistivity value so obtained is r ≈ 1 Ωcm. For Hall effect measurements, the samples were inserted between the poles of a permanent magnet system from ECOPIA (Republic of Korea), supplying a magnetic field B = 0.556 T. A negative value of the Hall coefficient was measured, indicating n-type conductivity. The resulting Hall mobility, $\mu =\frac{R_{H}}{\rho }\approx 50\;\frac{cm^{2}}{V\;s}$, is in agreement with values reported in the recent literature [47].

## 4. Gas Sensing Properties Characterization

### 4.1. Measurement Setup

The gas sensing performance of the manufactured sensors was characterized by means of a measurement setup described in detail by some of the authors in [30,48], and properly conceived to test chemoresistive gas sensors. The implemented measurement system allows researchers to monitor the variation of the sensing film conductance in the presence of different gas concentrations and species, and at different operating temperatures up to 300 °C in real-time, with a sampling time t_s_ = 300 ms in this work. During the tests, the sensor was placed in a stainless-steel measurement chamber where the gas flow was injected at a constant rate, set to 200 mL/min in this work. The gas flow was digitally controlled, allowing for the variance of the concentrations of target and carrier gases, while the total flow rate remained fixed by means of an accurate flowmeter system (BronkHorst F-201C) remotely controlled by a properly designed virtual instrument (VI) realized in LabView environment. The variation of the electrical conductance of the sensor was measured by a properly designed front-end electronics, embedding a biasing circuit for the chemoresistive sensor response readout as well as the conditioning electronics for monitoring temperature data coming from a platinum resistance temperature detector (Pt-RTD), screen printed on each sensor and for driving the sensor heater [30].

The functionalities of the front-end electronics (circuit biasing and signal acquisition) were managed by means of commercial data acquisition and conversion boards (National Instrument PXI-6351 and PXI 6713 boards).

The designed characterization system allowed for an accurate measurement of the temperature in the range between [120 °C and 400 °C], with an accuracy of 3 °C.

During tests, we measured the variations of the In_2_O_3−x_ sensor electrical resistance. The sensor response *R*_esp_ is defined as:

(11) $$Resp=\frac{R_{gas}-R_{0}}{R_{0}}$$

where *R_gas_* and *R*_0_ (baseline resistance) are the resistance of the film when exposed to the target gas (at the end of the exposure phase) and to the carrier gas (at steady state), respectively.

In this work, ultrapure air and nitrogen (N_2_) were used as carrier gases.

Gas sensing characterization follows a common pre-set measurement protocol. According to that, a pure carrier gas phase (air or N_2_) is followed by a target gas phase with different concentrations. In this work, three different NO_2_ concentrations were tested: 10 ppm, 5 ppm and 2.5 ppm. The measurement protocol started with a pure carrier gas phase with a duration of 4 min, followed by alternating gas phases, 8 min each, where the NO_2_ concentration was decreasing. The measurement closed after a 4-minute phase of pure carrier gas.

The repeatability obtained with the same sensor was evaluated around 5% of the response value during the six-month measurement campaign.

### 4.2. Experimental Results and Discussion

The In_2_O_3−x_ sensor was experimentally characterized in terms of baseline resistance and response to NO_2_ at different operative temperatures and carrier gases (oxygen-rich and oxygen-free carrier gas).

#### 4.2.1. Baseline Resistance

**Figure 5:**
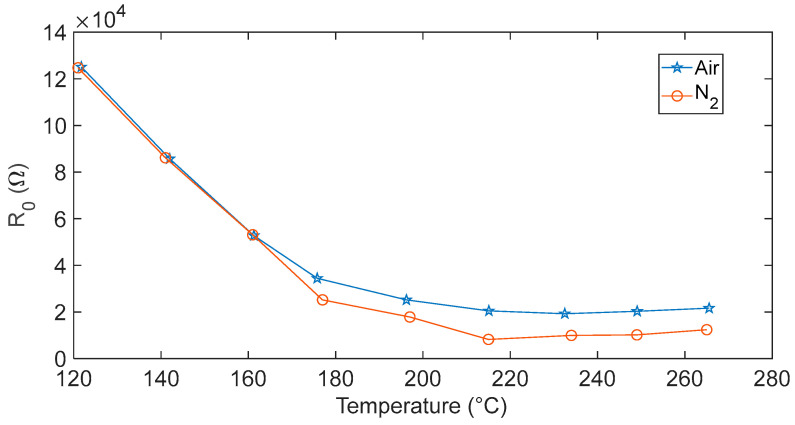
Measured baseline resistance of the In_2_O_3−x_ sensor evaluated at different temperatures in air (blue line) and N_2_ (red line) carrier gas.

The baseline resistance R_0_ of the sensor was measured in steady-state condition both in air and N_2_ at different operating temperatures, ranging from 120 °C to 260 °C. The measured resistance showed values comprised between 8 kΩ and 120 kΩ for both carrier gases used in this work. Figure 5 shows R_0_ measured in air and N_2_ as a function of the working temperature. Measured values were in accordance with the requirements of a low-cost measurement setup. As expected, the data confirm the semiconductor n-type behavior of the In_2_O_3−x_ film, showing an increased conductivity in N_2_ with respect to the one measured in air due to the donor type of the intrinsic defects. Indeed, the adsorption of the oxygen present in the atmosphere resulted in a negative charge trapped at the surface, a consequent increase in the inter-grain potential barrier and of the baseline resistance. Moreover, data show that the effect of the adsorbed oxygen was modest in the whole tested temperature range (a result which was also found in other works for In_2_O_3_ [26,28]). In particular, it was almost negligible for temperatures lower than 160 °C, where the chemisorption reactions in Equations (3) and (4) are not favored, and probably only physisorption occurs.

#### 4.2.2. Response to NO_2_

**Figure 6:**
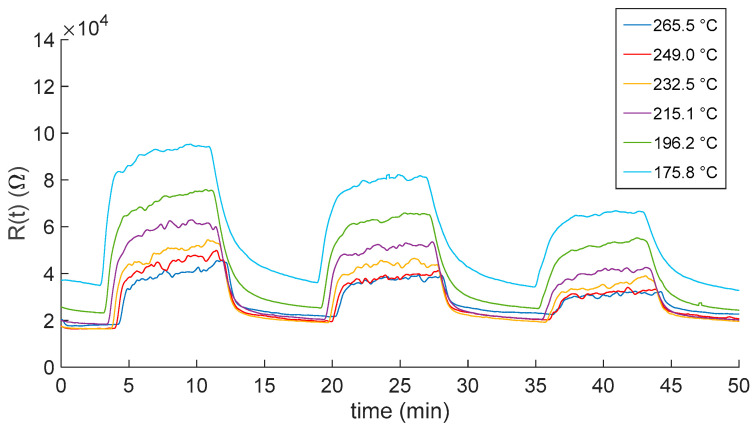
Measured In_2_O_3−x_ sensor response as a function of time when gas pulses (8 min long) consisting of mixtures of air and NO_2_ (10, 5 and 2.5 ppm) are injected into the measurement chamber. Gas pulses are followed by recovery phases (8 min long) in pure dry synthetic air. Different colors represent responses obtained at the different working temperatures (see legend).

**Figure 7:**
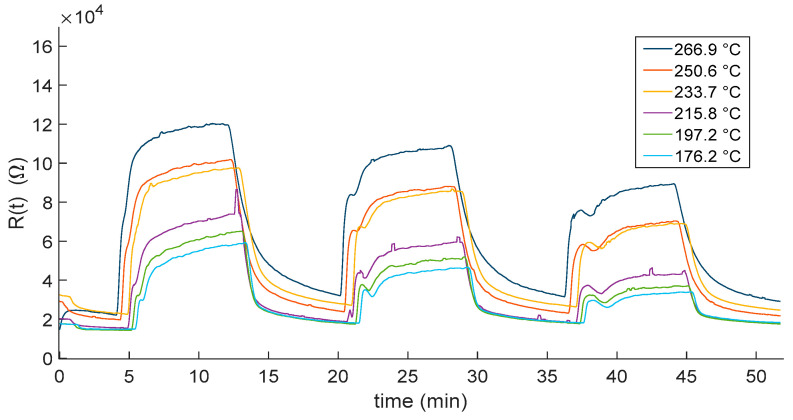
Measured In_2_O_3−x_ sensor response as a function of time when gas pulses (8 min long) consisting of mixtures of N_2_ and NO_2_ (10, 5 and 2.5 ppm) are injected into the measurement chamber. Gas pulses are followed by recovery phases (8 min long) in pure dry N_2_. Different colors represent responses obtained at the different working temperatures (see legend).

This section shows the experimental results related to the characterization of the sensor response to NO_2_. Figure 6 and Figure 7 show the transient responses to NO_2_ when air and N_2_ are used was carrier gases, respectively. Plots show the measured response as a function of time when exposing the sensors to mixtures with NO_2_ concentrations of 10 ppm, 5 ppm and 2.5 ppm. Each gas phase had a duration of 8 min, and it was followed by a recovery phase in which the sensors were exposed to the pure carrier gas. The whole measurement lasted approximately 50 min. The measurement protocol was repeated for different temperatures in the range 175–265 °C, as described in the plot legend.

As expected, the responses show that the film resistance decrease with increasing temperatures. Moreover, temperatures higher than 200 °C negatively affected the reactivity of the sensor. Indeed, for both the carrier gases, at the lower displayed temperatures, the sensor response did not reach the steady state, showing the need of a longer recovery time with respect to the one set for the measurement protocol (8 min). This may represent a limitation on the use of this kind of sensor in, e.g., alarm systems and environmental monitoring, where short recovery times are needed.

**Figure 8:**
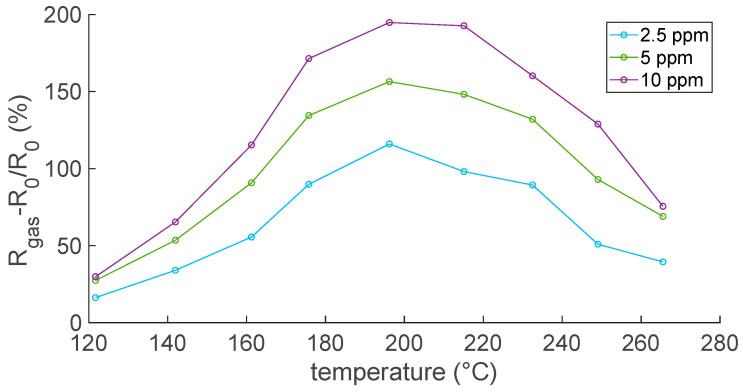
Responses to NO_2_ as a function of temperature; air is the carrier gas.

**Figure 9:**
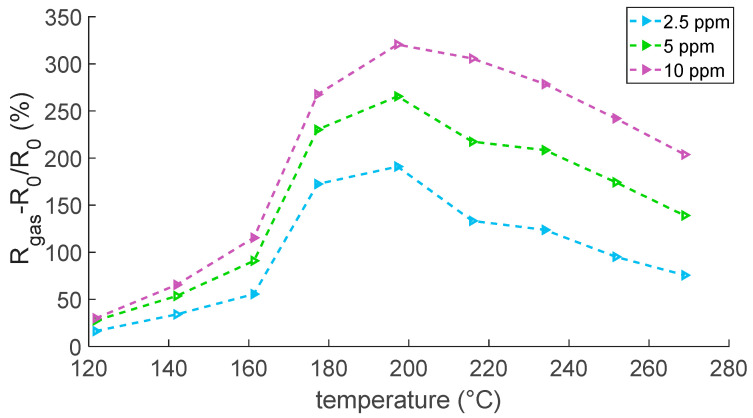
Responses to NO_2_ as a function of temperature; N_2_ is the carrier gas.

In this regard, the experimental results in Figure 8 and Figure 9 show that the response to NO_2_, evaluated according to Equation (8), increased with temperatures up to 200 °C, where the optimal operating temperature was reached, and then decreased. This is a typical behavior for MOX sensors.

In fact, chemosorption and ionosorption result from the combination of two elementary reaction steps (adsorption of neutral species form the gaseous phase and exchange of charge between these species and the solid). Adsorption is favored at lower temperatures [34,38] because the kinetic energy of the molecules makes the creation of weak electrostatic bonds on the solid surface more difficult, whereas the ionization of weakly bound neutral adsorbed species requires electrons to be thermally emitted from the Fermi energy across the surface barrier to become trapped in an adsorbed atom or in an adsorbed molecule. Since chemosorption and ionosorption are the combination of these two elementary reactions, there is an optimal temperature where the probability of occurrence becomes maximum.

**Figure 10:**
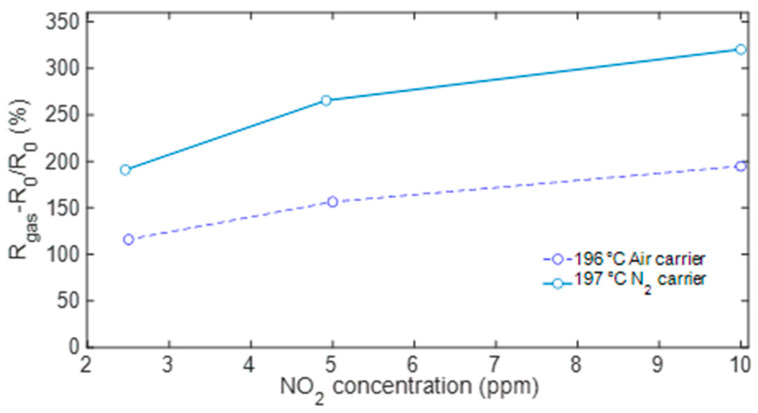
Sensor response to NO_2_ with air (solid line) and N_2_ (dashed line) as carrier gas evaluated at the optimal working temperature of 200 °C.

Finally, Figure 10 reports the sensor response as a function of the NO_2_ concentration: the plot compares results evaluated at the optimal temperature in air and N_2_.

The estimated response time, *t_res_,* and recovery time, *t_rec_*, evaluated using the transient responses shown in Figure 6 and Figure 7 at the optimal temperature are reported in Table 1. The response time reduced slightly when the gas concentration increased. As expected, the recovery time was almost independent of the gas concentration, and it was similar to the response time, differently from other materials and test conditions, for which it was significantly higher [38]. The response in the presence of oxygen was slightly slower than that in nitrogen. On the contrary, the recovery in the presence of oxygen was slightly faster than the one in nitrogen. This latter result cannot be explained if only reaction (1) is considered to describe the sensor behavior both in air and in nitrogen. Nevertheless, as already discussed, in air other reactions also play a role, especially at higher temperatures. In particular, the effect of reaction (5) could explain the faster recovery in air, since it justifies an additional release of electrons from the surface to the conduction band, which is due to the Interaction of adsorbed NO_2_ with adsorbed oxygen, forming adsorbed NO_3_^−^, and subsequently a different and possibly faster route for NO_x_ desorption. In all cases, on average, the response settled in about 2 min for all tested NO_2_ concentrations, i.e., within practical values for real-world applications.

**Figure 11:**
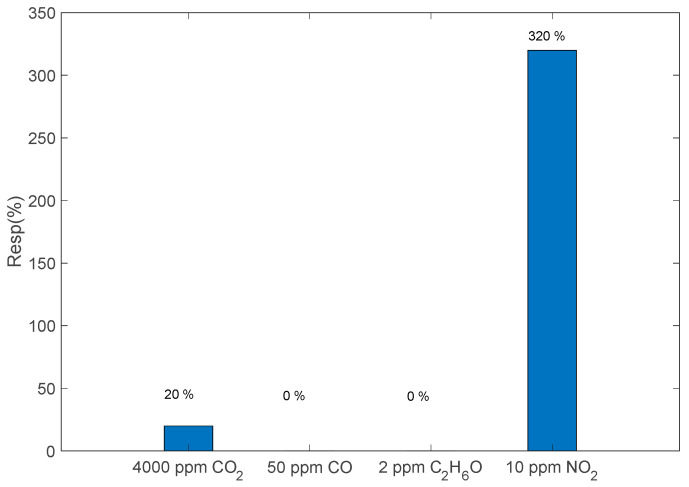
Sensor response to 4000 ppm CO_2_, 50 ppm CO and 2 ppm C_2_H_6_O compared with the response to 10 ppm NO_2_, according to Equation (1); N_2_ gas carrier, T ≈ 200 °C. Data evaluated by means of repeated transient gas measurements as for plots in Figure 3 and Figure 4.

Finally, the sensor cross-sensitivity to CO, CO_2_ and C_2_H_6_O was evaluated. CO and CO_2_ are among the most common interfering gases, with respect to NO_2_, involved in combustion processes, and the concentrations chosen for this evaluation are typical for exhaust gas emissions and are of interest for the applications for which the sensor was conceived. On the other hand, the cross-sensitivity to ethanol can give a good insight into the possible response to volatile organic compounds (VOCs). Reported data in Figure 11 refer to sensitivities evaluated at the optimal temperature. They show a satisfactory performance in terms of NO_2_ selectivity.

In order to investigate the effect of preabsorbed oxygen in the case of exposure to reducing gases, the same measurements were repeated with the interfering gases in air. The obtained results are similar to those observed in N_2_, confirming good selectivity behavior.

Lastly, the proposed sensors show performance comparable to, or even better, than those of thin-film metal oxide sensors recently reported in the literature, also in terms of selectivity [25,49,50,51,52,53] and, as highlighted in the comparison reported in Table 2, concerning pure MOX thin films.

## 5. Conclusions

In_2_O_3−x_-resistive sensors were manufactured by a magnetron sputtering deposition method at room temperature and in absence of oxygen gas on alumina printed circuit boards. Combined with the well-known uniqueness of this technique, the oxygen starvation during the film deposition allowed us to obtain layers with final resistance values in the order of kilohms, a convenient range for applications exploiting low-cost measurement systems and electronics. Moreover, this technique allowed us to speed up the procedure of film realization (it takes less than 1 h to realize multiple samples, half of the time experienced by the authors, i.e., in [26]), it ensured excellent adhesion to the substrate and it allowed us to improve the sensing response in terms of NO_2_ sensitivity. Indeed, the larger amount of oxygen vacancies characterizing the In_2_O_3−x_ surface facilitated the chemisorption reaction of the NO_2_ and enhanced the film conductivity. The response to NO_2_ of the In_2_O_3−x_-resistive sensors has been extensively characterized. The sensing performance of the realized sensor was measured at different NO_2_ concentrations in the range 2.5 ppm–10 ppm both with an oxygen-rich and oxygen-free atmosphere and at different operating temperatures. The tested sensor exhibited appreciable gas sensitivity in all the tested conditions, showing higher response in the case of an oxygen-free atmosphere. This is an important feature for highly required specific application scenarios, such as NO_2_ detection involved in the monitoring and control of fuel leaks in aerospace systems.

The sensors showed remarkable NO_2_ sensing capability, with a very high sensitivity evaluated as 32%/ppm to 10 ppm of NO_2_ in N_2_ and 20%/ppm to 10 ppm of NO_2_ in air at the optimal working temperature of 200 °C, which is a value comparable to, and even lower than, the working temperature of several thin-film MOX sensors available in the literature (Table 1, [19]). A sensitivity of 32%/ppm to 10 ppm of NO_2_ was evaluated in N_2_ with respect to a 20%/ppm to 10 ppm of NO_2_ in air at the optimal working temperature, evaluated as 200 °C. The observed responses show remarkable NO_2_ sensing capability, with a very high response at an operative working temperature that is in line with, and often even lower than, that of various thin-film MOX sensors available in the literature. The selectivity to CO_2_, CO and C_2_H_6_O gases was also investigated. The sensor exhibited no appreciable response, confirming the good performances for NO_2_ detection and, in particular, in combustion gas condition monitoring applications.

## Figures and Tables

**Table 1:** Response time, *t_res_*, and recovery time, *t_rec_*, of In_2_O_3−x_ sensor to three different NO_2_ concentrations evaluated at the optimal working temperature of 200 °C.

Concentration (ppm)	*t_res_* (min)	*t_rec_* (min)
10 ppm (Air carrier)	1.45	1.84
5 ppm (Air carrier)	1.68	1.88
2.5 ppm (Air carrier)	1.82	2.03
10 ppm (N_2_ carrier)	1.14	1.90
5 ppm (N_2_ carrier)	1.29	1.98
2.5 ppm (N_2_ carrier)	1.98	2.27

**Table 2:** Sensing performance of MOX-based thin film sensors.

Material	Technology	NO_2_ Concentration (ppm)	Operating Temperature (°C)	Response (%)	Response/ Recovery time	Reference
WO_3_	Reactive ion magnetron sputtering	3	157	650	200 s/-	[54]
WO_3_	PVD	5	130	6000	100 s/300 s	[55]
ZnO		200	450	94	162 s/134 s	[56]
ZnO	DC magnetron sputtering + laser lithography	4	175	600	-/-	[57]
NiO	RF magnetron sputtering	350	150	82	90 s/120 s	[58]
CuO	CVD	4.5	200	220	51 s/124 s	[59]
In_2_O_3_	RF magnetron sputtering	1	150	91	195 s/285 s	[12]
In_2_O_3_	Pulsed electron deposition	24	300	600	~180 s/~180 s	[26]
In_2_O_3−x_	RF magnetron sputtering	10	200	320	90 s/110 s	This work
SnO_2_/NiO	DC magnetron sputtering	10	200	144	37 s/98 s	[60]

## Data Availability

Not applicable.
